# Efficacy and safety of allogeneic umbilical cord-derived mesenchymal stem cells for the treatment of complex perianal fistula in Crohn’s disease: a pilot study

**DOI:** 10.1186/s13287-023-03531-0

**Published:** 2023-10-31

**Authors:** Juan Wei, Yufei Zhang, Chunyan Chen, Xiaoyue Feng, Zhao Yang, Jing Feng, Qiong Jiang, Jinjin Fu, Ji Xuan, Hong Gao, Lianming Liao, Fangyu Wang

**Affiliations:** 1 Department of Gastroenterology and Hepatology, Jinling Hospital, Affiliated Hospital of Medical School of Nanjing University, No. 305 East Zhongshan Road, Nanjing, People’s Republic of China; 2 Department of General Surgery, Jinling Hospital, Affiliated Hospital of Medical School of Nanjing University, No. 305 East Zhongshan Road, Nanjing, People’s Republic of China; 3 Department of Gastroenterology and Hepatology, The First School of Clinical Medicine, Southern Medical University, Guangzhou Da Dao Bei 1838, Guangzhou, China; 4 Department of Gastroenterology and Hepatology, The Affiliated Changzhou No. 2 People’s Hospital, Nanjing Medical University, Changzhou, China; 5 Department of Radiology, Jinling Hospital, Affiliated Hospital of Medical School of Nanjing University, No. 305 East Zhongshan Road, Nanjing, People’s Republic of China; 6 Center of Laboratory Medicine, Union Hospital of Fujian Medical University, No. 29, Xinquan Road, Fuzhou, 350001 People’s Republic of China

**Keywords:** Perianal fistula, Crohn’s disease, Umbilical cord, Mesenchymal stem cell

## Abstract

**Objectives:**

The aim of the study was to evaluate the efficacy and safety of allogeneic umbilical cord-derived mesenchymal stem cells (TH-SC01) for complex perianal fistula in patients with Crohn’s disease (CD).

**Methods:**

This was an open-label, single-arm clinical trial conducted at Jinling Hospital. Adult patients with complex treatment-refractory CD perianal fistulas (pfCD) were enrolled and received a single intralesional injection of 120 million TH-SC01 cells. Combined remission was defined as an absence of suppuration through an external orifice, complete re-epithelization, and absence of collections larger than 2 cm measured by magnetic resonance imaging (MRI) at 24 weeks after cell administration.

**Results:**

A total of 10 patients were enrolled. Six patients (60.0%) achieved combined remission at 24 weeks. The number of draining fistulas decreased in 9 (90.0%) and 7 (70.0%) patients at weeks 12 and 24, respectively. Significant improvement in Perianal Crohn Disease Activity Index, Pelvic MRI-Based Score, Crohn Disease Activity Index, and quality of life score were observed at 24 weeks. No serious adverse events occurred. The probability of remaining recurrence-free was 70% at week 52.

**Conclusion:**

The study demonstrated that local injection of TH-SC01 cells might be an effective and safe treatment for complex treatment-refractory pfCD after conventional and/or biological treatments fail (ClinicalTrials.gov ID, NCT04939337).

*Trial Registration*: The study was retrospectively registered on www.ClinicalTrials.gov (NCT04939337) on June 25, 2021.

## Introduction

Crohn’s disease (CD) is a complex, chronic disorder that primarily affects the digestive system. CD involves an abnormal immune response that causes excess inflammation. Anal fistula is the most common complication of CD [1]. CD perianal fistula (pfCD) is often treatment refractory, with frequent recurrences. pfCD imposes a significant economic burden on patients and impairs their quality of life. Treating pfCD requires multidisciplinary teamwork combining medication and surgery [2]. Recently biological agents, such as Infliximab, Certolizumab pegol, and Ustekinumab, have achieved a high rate of fistulae closure [3]. However, recurrence is still a challenge. The closure rate of pfCD after Infliximab treatment was 63% at week 14, but decreased to 36% at week 54 [4]. For ustekinumab, the closure rate was roughly 40% at 6 months [5]. Remission can only be achieved in half of the cases even with optimal surgical and biological therapy [5, 6].

Mesenchymal stromal cells (MSCs) are defined as a plastic-adherent cell population that can be induced to differentiate in vitro into cells of osteogenic, chondrogenic, and adipogenic lineages. MSCs can exert immunomodulatory and anti-inflammatory function by secreting a variety of bioactive molecules and regulating local immune response [7, 8]. In 2003, Garcia-Olmo reported autologous MSCs were effective for recurrent rectovaginal fistula in a female CD patient [9]. In March 2018, allogeneic, expanded, adipose-derived stem cells (Darvadstrocel) were approved by the European Medicines Agency for the treatment of complex perianal fistulas in adult patients with non-active/mildly active luminal CD [10].

Although MSCs can be readily harvested from adipose tissue, liposuction is an invasive surgical procedure. Umbilical cord (UC) is an attractive alternative to adipose. Compared to MSCs derived from adult adipose, UC-MSCs may be cultured longer and have higher proliferation capacity [11]. A substantial number of UC-MSCs can be produced after several passages. Isolation of UC-MSCs is ethically acceptable as UC is considered to be medical waste. Moreover, UCB-MSCs exhibited a greater anti-inflammatory effect than adipose-derived MSCs [12–14].

We conducted a single-arm, pilot study to evaluate the safety and effects of TH-SC01, an investigational, allogeneic, umbilical cord-derived, off-the-shelf MSC product candidate, to treat patients with pfCD that was refractory to conventional and/or biological treatments. We hypothesized that UC-MSCs locally injected around perianal fistulas were safe and effective at the end of the 24-week follow-up and could increase the health-related quality-of-life scores.

### Patients and methods

**Fig. 1:**
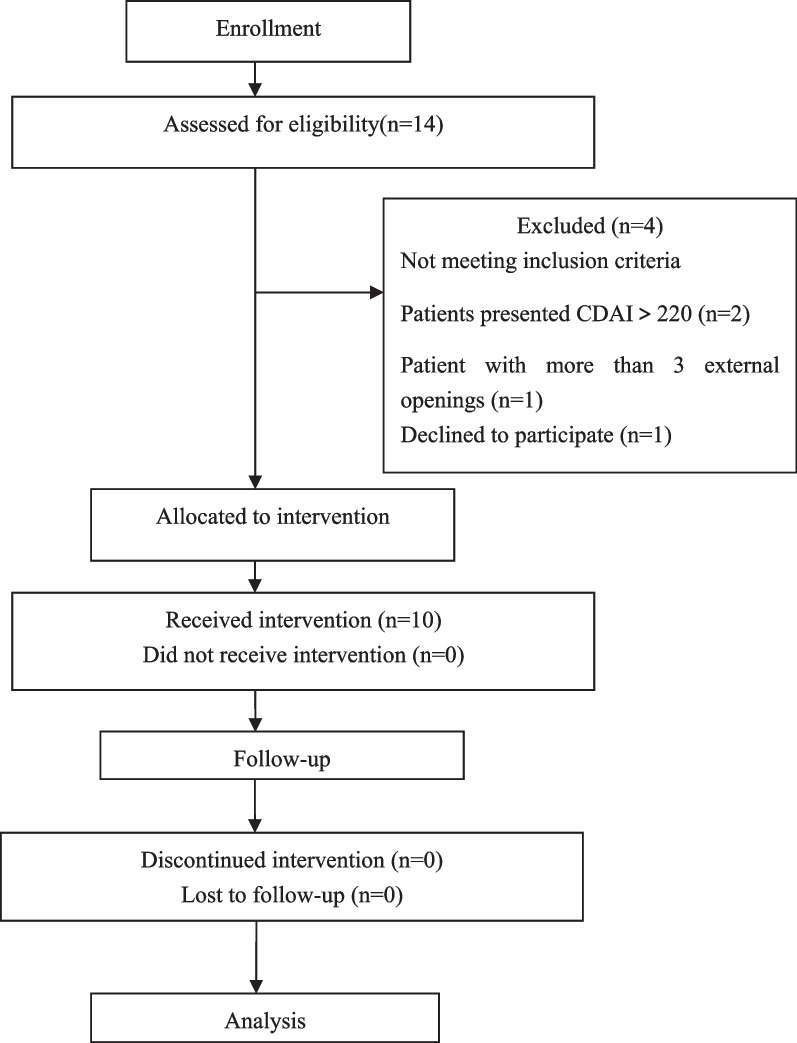
Flowchart of the clinical trial

This was a prospective single-arm pilot study conducted at Jinling Hospital, a tertiary care academic hospital in Nanjing. Patients were enrolled from November 2020 to November 2021. We included patients who were 18 years of age or older and fulfilled the Committee of the American Society of Colon and Rectal Surgeons 2016 diagnostic criteria for complex pfCD; and had non-active or mildly active luminal CD defined as Crohn’s Disease activity index (CDAI) < 220, with a minimum duration of 6 months. In addition, they had to have received surgical therapy (drainage or seton placement) and systemic administration of anti-TNF-alpha for at least 14 weeks. Patients with more than 1 internal and 3 external openings, abscess, stenosis, severe proctitis, active infections or lymphoproliferative active diseases were excluded. Patients who were allergic to anesthetics or magnetic resonance image scan (MRI) contrast were also excluded. The clinical trial process is shown in Fig. 1.

### Production of GMP-grade human UC-MSCs

TH-SC01 is a cellular product manufactured in a Good Manufacturing Practice (GMP) facility at TopCel-KH Biopharmaceutical (Nanjing, China). Umbilical cord tissue was donated by a healthy volunteer whose baby was delivered by elective cesarean section. Informed consent was obtained before cesarean section.

Briefly, cord tissue was placed in a sterile bottle containing DMEM/F12 and transported to the GMP facility. After umbilical vessels were removed, Wharton’s jelly was sliced into fragments of about 1 cm in length. The fragments were placed on culture plates in DMEM/F12 media with 1% platelet lysate (AventaCell BioMedical Corp, Atlanta, USA). After about 15 days, cord fragments were discarded and cells were digested with GMP-grade enzymes (Gibco Life Technologies, Waltham, USA). Cells were subsequently passaged. UC-MSCs of passage 3 were cryopreserved in cryovials in the vapor phase of liquid nitrogen as master cell bank (MCB). For quality control, karyotyping and viral tests were performed. Karyotype analysis confirmed a normal human karyotype. UC-MSCs were also evaluated for cell surface markers CD44, CD73, CD90, CD105, CD31, CD34, and CD45. We thawed cells for subculture under the same conditions. Cells cultured to passage 5 were used in the present study.

The final cells were harvested into a solution with 5% human serum albumin. Release testing included total nucleated cell count, viability, endotoxin, and pathogens (human immunodeficiency virus-1 and -2, cytomegalovirus, hepatitis B virus, hepatitis C virus, human T lymphocytic virus, Epstein-Barr virus, and mycoplasma). Pathogens were detected using reverse transcriptase PCR. For each patient, 120 million cells were formulated in 24 mL and supplied in four vials (6 mL/vial). Cells were shipped to the hospital on the day of administration. The formulated product can be stored for a maximum of 48 h between 4 and 8 °C.

### Treatment procedure

A pelvic MRI scan was performed at the screening to guide the surgical procedure and to assess abscesses. Two weeks before cell administration, patients might be treated with fistula curettage and seton placement under anesthesia.

**Fig. 2:**
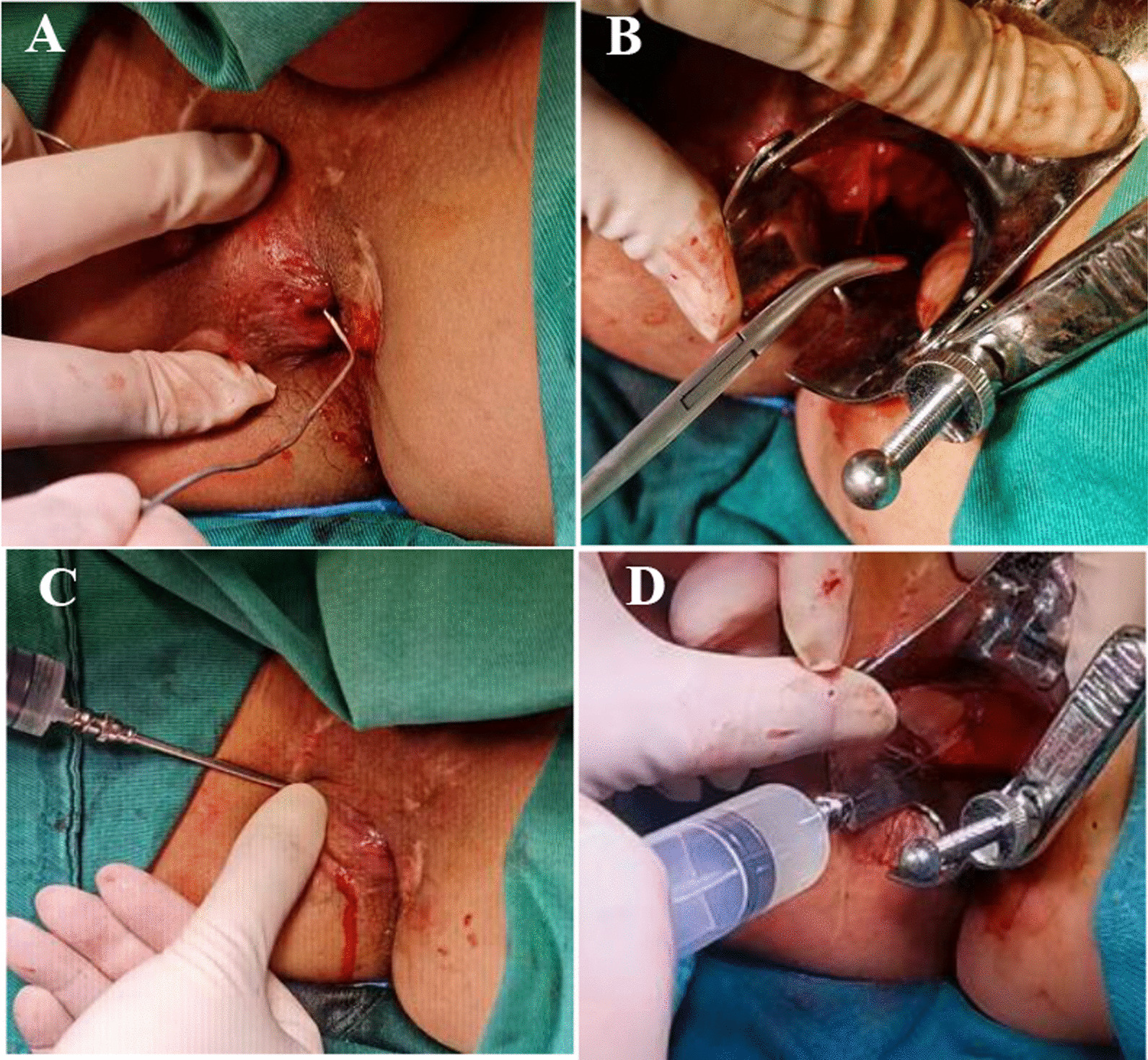
Treatment procedure. (**A**) Curettage was performed after the internal orifice was identified. (**B**) The internal orifice was closed and confirmed. (**C**) Tightness of the seal was checked through the external opening. (**D**) UC-MSC solution was injected around the IO and tracts

If a seton was placed, it was withdrawn immediately before cell administration. Closure of the internal opening was achieved using polyglactin absorbable 2/0 stitches and confirmed by injection of 10 mL saline solution through the external opening. Subsequently, 120 million cells were injected with a 22G needle into tissues adjacent to all fistula tracts and the internal opening. This dose was selected according to the studies with Darvadstrocel. Half of the cells were injected via the anal canal into the tissues surrounding the sutured internal opening, and the other half of cells were injected through the external opening (s) into the fistula walls (no deeper than 2 mm) along the fistula tract (s) (Fig. 2).

During the study, patients could be treated with antibiotics. Immunomodulators and anti-TNF mAbs were maintained at stable doses. A steroid course was permitted to treat occurrences of luminal disease during the study, with a starting dose of 40 mg tapered over a maximum of 12 weeks.

Patients were followed up at weeks 1, 2, 4, 12, 24 and 52. Outcomes were assessed by masked investigators at baseline and all study visits. Pelvic MRI was performed at 12 weeks and 24 weeks [15].

### Outcomes

The primary endpoint was combined remission at weeks 24, defined as no draining in all treated external openings despite gentle finger compression, and the absence of collections larger than 2 cm of the treated perianal fistulas in at least two of three dimensions by MRI.

There were two key secondary endpoints: clinical remission and clinical response. Clinical remission was defined as closure of all treated external openings that were draining at baseline by week 24. Clinical response was defined as closure of at least 50% of all treated external openings that were draining at baseline by week 24.

Other secondary endpoints were Perianal Crohn’s Disease Activity Index (PDAI)/CDAI score, VAS score for pain, quality of life by the Inflammatory Bowel Disease Questionnaire (IBDQ), and MRI-based Van Assche score at weeks 12 and 24.

We monitored adverse events, particularly exacerbations of symptoms, using a standard adverse-event case report form at each visit.

### Statistical analysis

Categorical variables were described as frequencies, percentages, and ordinal and continuous variables, with the median and min–max range. The Wilcoxon test was used to compare the baseline and final results of the PDAI, Van Assche score, CDAI, and IBDQ. A two-sided *P* value of less than 0.05 indicated statistical significance.

## Results

### Baseline characteristics of the patients

Fourteen patients were screened and ten were enrolled. The average age was 34.3 years (range 22–53). Eight were male. Mean durations of CD and fistula were 5 years (range 2–15) and 4 years (range 1–12), respectively. The numbers of fistula tracts were one (*n* = 3), two (*n* = 6), and three (*n* = 1), respectively, and the numbers of external openings were one (*n* = 4), two (*n* = 5), and three (*n* = 1), respectively (Table 1).

**Table 1:** Demographic and baseline characteristics

Variable	UC-MSCs (*n* = 10)
Male (%)	8 (80)
Age (years)	34.3 (22–53)
Median BMI (range)	22(18–23)
Median CD duration, y (range)	8(2–18)
Median fistula duration, y (range)	5(1–16)
History of surgery for fistula, n (%)	7 (70)
*Fistulas prior treatment failure*	
Antibiotics	8
Anti-TNF	2
*Number of fistula tracks*	
1	3
2	6
3	1
*Number of external openings*	
1	4
2	5
3	1
*Location*	
Extra-sphincteric	2
Inter-sphincteric	5
Supra-sphincteric	2
Trans-sphincteric	1
Extension	
Infraelevator	6
Supraelevator	4
*Rectum wall involvement*	
Normal	3
Thickened	7

### Clinical outcomes

**Fig. 3:**
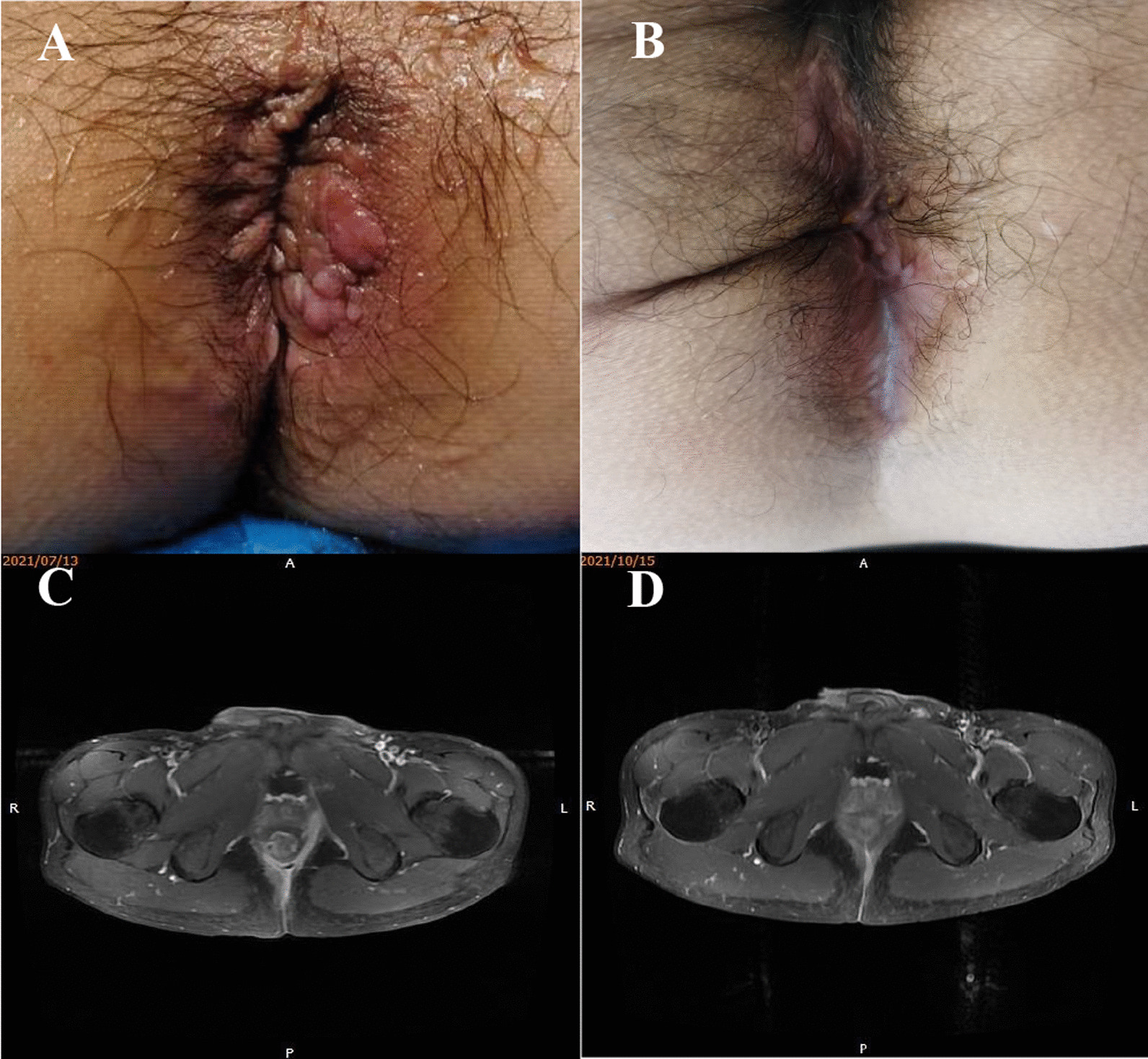
Patient representative of combined remission (male, 34 years old). (**A**, **B**) Photograph of the external fistula orifice before and 12 weeks after treatment. (**C**, **D**) MRI before and 12 weeks after treatment

Sixty percent (6/10) of patients achieved combined remission at 24 weeks. Ninety percent (9/10) and seventy percent (7/10) of patients had a clinical response at 12 weeks and 24 weeks, respectively (Table 2). Two patients had complete occlusion of fistula tracts on MRI at 24 weeks (Fig. 3). Seven patients with clinical response at 24 weeks did not recur at 52 weeks.

**Table 2:** Efficacy results

Variable	*n*
*Closure of external openings after 12 weeks*	
No	1
Yes (at least 1)	9
*MRI absence of collections > 2 cm after 12 weeks*	
No	1
Yes (at least 1)	9
*Closure of external openings after 24 weeks*	
No	3
Yes (at least 1)	7
*MRI absence of collections > 2 cm after 24 weeks*	
No	3
Yes (at least 1)	7
*Subjects presenting luminal relapse after 24 weeks*	
No	9
Yes	1

There were 17 external fistulas in 10 patients, and 13 were closed and epithelialized at 12 weeks. The external fistula recurred in two patients at 24 weeks (Table 2).

**Fig. 4:**
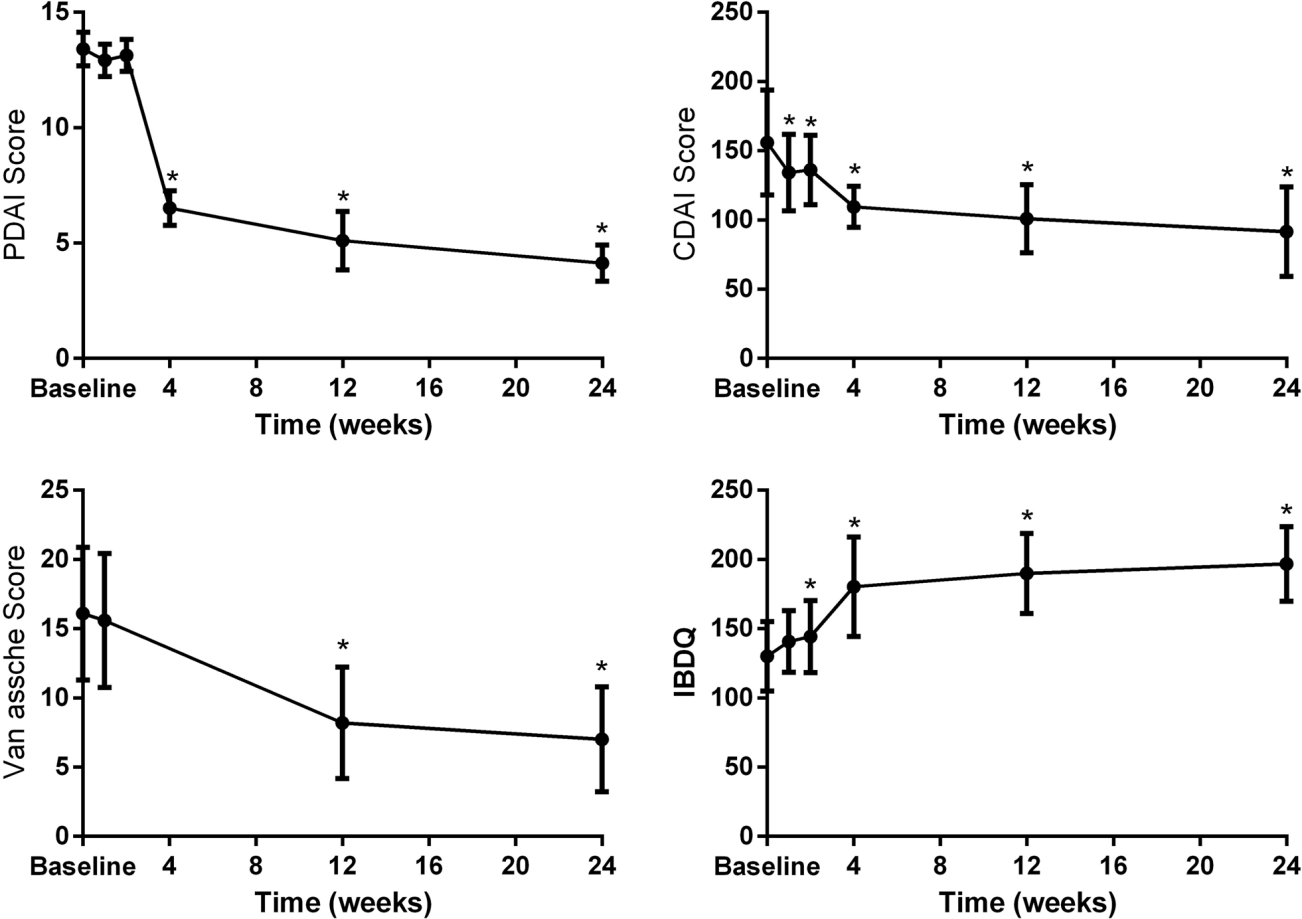
Treatment outcomes at different time points. PDAI: Perianal Crohn’s Disease Activity Index, IBDQ: Inflammatory Bowel Disease Questionnaire. Van Assche score (Pelvic MRI-Based Score). A two-sided P value of less than 0.05 indicated statistical significance: Wilcoxon signed rank test adjusted for multiplicity

Overall, a significant reduction of the PDAI score was observed after MSCs administration. Van Assche scores demonstrated a significant reduction from the 12th week. Although CDAI scores were all less than 220, the reduction was also significant. In addition, IBDQ increased during follow-up (Fig. 4).

During the study, no patient received steroids for a flare of CD. No patient received antibiotics or anti-TNF treatment as a rescue treatment for fistula.

### Safety

Three patients had adverse events, which were perianal pain and low fever, elevated liver enzymes, and perianal pain, respectively. All adverse events were resolved within a week.

## Discussion

Symptomatic pfCD is a devastating complication. In this pilot study, the number of draining fistulas decreased in most patients (90%) at 12 weeks after UC-MSCs administration. More than half of patients (60%) showed closure of all treated external openings at 24 weeks and did not recur during the 1-year follow-up. All patients reported significantly improved quality of life.

As pfCD is characterized by increased inflammation that impedes epithelial healing [16, 17], the underlying mechanism of MSCs might mainly involve their immunomodulatory ability. MSCs are capable of regulating the functions of a diverse array of cells in the immune system. MSCs can interact with B lymphocytes, T lymphocytes, NK cells, monocyte-derived dendrite cells, and neutrophils, affecting differentiation and activation of these immune cells [18, 19]. In animal models, MSCs have been shown to inhibit production of a wide panel of inflammatory cytokines and chemokines and increased interleukin-10 levels, directly acting on activated macrophages [20]. These pre-clinical data are consistent with the observed clinical outcomes with MSCs for pfCD and CD [8–10].

The preliminary results showed that UC-MSCs might be superior to adipose-derived MSCs (AD-MSCs) at the same cell dose [21]. In the phase 3 trial, Panés et al. reported that the combined remission rate was 50% (53/107) for AD-MSCs, while it was 60% (6/10) in our study [22]. Although MSCs from different tissues have similar surface antigen expression, their biological attributes are different, especially their immunomodulatory activity. For example, Najar et al. showed that in mixed leukocyte reactions (MLRs) using irradiated allogeneic peripheral blood mononuclear cells (PBMCs) as stimulating cells to activate T-lymphocytes, MLR suppression by UC-MSCs was more extensive than by MSCs from both bone marrow and adipose tissue [23]. UC-MSCs secreted greater amount of leukemia inhibitory factor than did AD-MSCs. [24, 25] Similarly, Li et al. reported that PHA/IL-2-induced T cell proliferation was suppressed more intensively by UC-MSCs than by AD-MSCs [14]. Ayatollahi et al. reported UC-MSCs caused greater growth reduction of PBMCs than did AD-MSCs when they were co-cultured with allogeneic phytohemagglutinin (PHA) activated PBMCs [13]. Thus, compared with AD-MSCs, human UC-MSCs have a greater ability for immune regulation. Additionally, UC-MSCs have higher proliferation and colony-forming potential [26]. Therefore, UC-MSCs might be the preferred choice of stem cells for anal fistula.

Although anti-TNF monoclonal antibody treatment provides a new option for the management of Crohn’s fistula [4], some patients cannot achieve complete healing. In a clinical study with infliximab, only 36 percent of patients in the infliximab maintenance group had a complete absence of draining fistulas at week 54 [27]. In addition, there was a high recurrence rate during maintenance therapy [28]. The sustained response in this study seems to be greater than that of the previous reports with anti-TNF monoclonal antibody [29–31]. In our study, patients who failed to Infliximab for more than 12 weeks were enrolled and MSCs still showed a promising outcome. Given that recurrence is common in patients with pfCD, our study has important implications. It is worth noting that fistula exudation reappeared in two patients at 24 weeks, who showed closure of all treated external openings at week 12. We propose that a second dose of MSCs might improve the long-term effects.

We found the improvement of MRI score was not parallel to remission of clinical symptoms. Clinical symptoms began to alleviate about 7 days after cell injection, while significant improvement shown by MRI only appeared about 12 weeks later. We think this is because the hole between two openings needs longer time to be filled by new tissue than the time that closure of the openings needs. Therefore, follow-up MRI exanimation may be performed after 12 weeks.

Most recently, the American Society of Colon and Rectal Surgeons has published clinical practice guidelines on the management of anorectal abscess, fistula-in-ano, and rectovaginal fistula and recommends that for select patients with Crohn’s disease who have refractory anorectal fistulas, local treatment with MSCs is considered effective and safe [32]. The recommendation was based on several phase I, phase II, and phase III clinical trials with bone marrow-derived and adipose-derived MSCs [22, 33–38]. Our study provides further evidence that MSCs are safe and effective for pfCD. The optimal therapeutic goals for the treatment of Crohn’s fistulae are complete healing of the lesions without recurrence and no injury to the anal sphincter. Surgery including incision, drainage and placement of loose (draining) setons may result in fecal incontinence ranging from 30 to 40% [29, 39]. In this context, MSC therapy could be a promising treatment option for pfCD refractory to conventional therapy.

## Conclusion

In conclusion, our preliminary findings demonstrated local UC-MSCs treatment produced a favorable therapeutic outcome and had a good safety profile in patients with pfCD for longer than 24 weeks. Randomized controlled trials involving larger clinical samples are warranted to assess the generalizability of our findings. Also, more mechanical studies are needed to deepen our understanding of this promising therapy.

## Data Availability

The datasets of the current study are available from the corresponding authors on reasonable request.

## Abbreviations

CD: Crohn’s disease
pfCD: CD perianal fistulas
MRI: Magnetic resonance image
MSCs: Mesenchymal stromal cells
UC: Umbilical cord
CDAI: Crohn’s disease activity index
PDAI: Perianal Crohn’s disease activity
GMP: Good manufacturing practice
MCB: Master cell bank
IBDQ: Inflammatory bowel disease questionnaire
MLRs: Mixed leukocyte reactions
PBMCs: Peripheral blood mononuclear cells
PHA: Phytohemagglutinin
